# Phylogeography and virulence structure of the powdery mildew population on its 'new' host triticale

**DOI:** 10.1186/1471-2148-12-76

**Published:** 2012-06-01

**Authors:** Veronique Troch, Kris Audenaert, Boris Bekaert, Monica Höfte, Geert Haesaert

**Affiliations:** 1 Associated Faculty of Applied Bioscience Engineering, University College Ghent, Valentin Vaerwyckweg 1, B-9000, Ghent, Belgium; 2 Department of Crop Protection, Faculty of Bioscience Engineering, Ghent University, Coupure links 653, B-9000, Ghent, Belgium

**Keywords:** Renal cell carcinoma, Partial nephrectomy, Robotic partial nephrectomy, Oncologic outcomes, Nephron-sparing surgery

## Abstract

**Background:**

Powdery mildew, caused by the obligate biotrophic fungus *Blumeria graminis*, is a major problem in cereal production as it can reduce quality and yield. *B. graminis* has evolved eight distinct *formae speciales* (f.sp.) which display strict host specialization. In the last decade, powdery mildew has emerged on triticale, the artificial intergeneric hybrid between wheat and rye. This emergence is probably triggered by a host range expansion of the wheat powdery mildew *B. graminis* f.sp. *tritici*. To gain more precise information about the evolutionary processes that led to this host range expansion, we pursued a combined pathological and genetic approach.

**Results:**

*B. graminis* isolates were sampled from triticale, wheat and rye from different breeding regions in Europe. Pathogenicity tests showed that isolates collected from triticale are highly pathogenic on most of the tested triticale cultivars. Moreover, these isolates were also able to infect several wheat cultivars (their previous hosts), although a lower aggressiveness was observed compared to isolates collected from wheat. Phylogenetic analysis of nuclear gene regions identified two statistically significant clades, which to a certain extent correlated with pathogenicity. No differences in virulence profiles were found among the sampled regions, but the distribution of genetic variation demonstrated to be geography dependent. A multilocus haplotype network showed that haplotypes pathogenic on triticale are distributed at different sites in the network, but always clustered at or near the tips of the network.

**Conclusions:**

This study reveals a genetic structure in *B. graminis* with population differentiation according to geography and host specificity. In addition, evidence is brought forward demonstrating that the host range expansion of wheat isolates to the new host triticale occurred recently and multiple times at different locations in Europe.

## Background

Genetic and archaeological evidence indicates that cereal agriculture, including wheat (*Triticum aestivum*) and rye (*Secale cereale*) cultivation, originated in the Fertile Crescent 10,000 to 12,000 years ago [1,2]. Since then, the nature of the agro-ecosystem itself has played a critical part in the emergence and spread of plant pathogens, by providing a more dense and genetically uniform host population [2]. An example of such a plant pathogen is *Blumeria graminis*, the causal agent of powdery mildew in cereals. This pathogen is a major problem in cereal production as it can reduce quality and yield [3,4]. Management of the disease normally involves the use of fungicides, but this adds considerably to the cost of grain production.

*B. graminis* is an obligate biotrophic fungus which implies that it depends on living plant cells for survival and reproduction. Penetration and biotrophic colonization of a plant is the result of highly specific recognition mechanisms in the gene-for-gene response involving avirulence (*AVR*)- and resistance genes [5]. Most known *AVR* genes encode effectors that facilitate pathogenicity by suppressing pathogen-associated molecular pattern-triggered immunity which can result in the induction of effector-triggered immunity [6,7]. Two *AVR* genes have been isolated from *B. graminis* and both have effector activity [8,9]. The fungus also has a huge repertoire of homologues of these genes, which may function as effectors and contribute to parasite aggressiveness [10].

In the long run, this mutual close interplay can result in co-evolution of host and pathogen. In this way, *B. graminis* has evolved eight distinct *formae speciales* (f.sp.) which display strict host specialization [11,12]. For example, *Triticum* spp. and *Secale* spp. are appropriate hosts to *B.g.* f.sp. *tritici* and *B.g.* f.sp. *secalis*, respectively. Conversely, if plants are attacked by inappropriate *formae speciales*, attempts to infect and colonize the plant will fail [13].

Multilocus phylogenetic analysis identified lineages, closely matching the *formae speciales* defined on the basis of host specialization [14-16]. Additionally, Sacristan et al. [10] found that *B. graminis* avirulence genes (*AVR*_k1_ paralogs) have evolved differentially in the different *formae speciales* and that the phylogeny of *AVR*_a10_-like sequences corresponds with those of other genes. In particular, *B.g.* f.sp. *tritici* and *B.g.* f.sp. *secalis* are phylogenetically the most closely related *formae speciales*.

In the last decade, several studies tried to tackle the co-evolutionary relationship of the different *formae speciales* with their hosts [14,15,17,18]. These studies resulted in a wide range of divergence time estimates between the different *formae speciales*, which reflects the challenges to calculate an accurate divergence time of this pathogen. However, all these studies state that the divergence of the different *formae speciales* is likely to be younger than the divergence of their hosts. This type of co-evolution is called host-tracking in contrast to co-speciation, where host and pathogen have diverged simultaneously [2]. In spite of the discrepancy between divergence time of host and pathogen, Wyand and Brown [14] concluded that the center of diversity of *B. graminis* coincides with the center of origin of their hosts in the Middle East.

Triticale (× *Triticosecale* Wittmack) is the intergeneric hybrid between the female parent wheat (*Triticum* ssp.) and the male parent rye (*Secale* ssp.). The aim of this hybrid breeding was to combine the cold and disease tolerance of rye and its adaption to unfavorable soils and climates with the productivity and nutritional qualities of wheat [16]. This artificial cereal is, in contrast to wheat and rye, of very recent origin and was commercialized at the end of the 1960’s [19,20]. During the last decade, triticale has gained considerable importance in Europe, as its production area has nearly doubled since 2000 (up to almost 3.6 million ha in 2009, [21]). However, with the expansion of the triticale growing area, powdery mildew emerged on this new host and became a significant disease on triticale. This was simultaneously observed in several European countries, including Belgium, France and Poland [16]. Recent research demonstrated that this powdery mildew on triticale has emerged most probably through a host range expansion of the wheat powdery mildew *B.g.* f.sp. *tritici*[16]. This means that powdery mildew has evolved the capacity to colonize a new host species, triticale, which is phylogenetically closely related to its present host wheat.

Host range expansions are expected to occur rather frequently, because only a few modifications in the effector repertoire of the pathogen can suffice to adapt to a closely related plant species [22]. Moreover, pathogen species with a large and diversified composition of effectors, which holds true for powdery mildew [23], should have a higher chance to increase their host range [22].

This study was initiated to gain more precise information about the evolutionary processes that led to this host range expansion. To address this question, we pursued a combined pathological and genetic approach. Powdery mildew isolates on triticale, wheat and rye were sampled from different breeding areas in Europe. Old isolates from the Middle East, the proposed centre of origin of cereals and *B. graminis,* were also included in this study, in order to be able to put the results in an evolutionary perspective. The first objective was to assess the host specificity and to determine the virulence profile of the powdery mildew populations on different hosts and regions in Europe. Documenting the true host range and virulence profile of this recently emerged pathogen is an important first step towards understanding the ecological context in which it has evolved. The second objective was to clarify the phylogenetic relationships and population structure of this recently emerged pathogen. We hypothesized that (i) the triticale powdery mildew population is structured by geography, host species or both and (ii) genetic diversity is higher in the wheat powdery mildew populations than in the recently emerged triticale powdery mildew populations.

## Results

### Host specialization

Pathogenicity tests were performed to assess host specificity of isolates of the recently emerged powdery mildew on triticale. A total of 72 isolates from triticale (35), wheat (35) and rye (2) were inoculated onto different triticale, wheat and rye cultivars (Table 1, Table 2). All isolates formed mildew colonies and sporulated abundantly on their respective hosts (Table 3, Additional files 1 and 2). All 35 isolates collected from triticale were able to infect several wheat cultivars, although they were less aggressive than isolates collected from wheat. In contrast, only 5 out of 35 isolates collected from wheat were pathogenic on triticale. None of the 70 isolates collected from wheat and triticale were pathogenic on rye. Isolates collected from rye were not able to infect wheat, but were pathogenic on 2 (out of 15) triticale cultivars (Grandval and Tribeca).

**Table 1:** ***Blumeria graminis*isolates included in the analysis**

**Isolate code**	**Source host (origin of isolates)**	**Sampling region**	**Location**	**Number of isolates**	**Year of collection**
Bgta_A(1-20)	*Triticum aestivum*	A	Belgium	39	2009–2010
BgTR_A(21-37)	triticale				
BgS_A(38-39)	*Secale cereale*				
Bgta_B(1-7)	*Triticum aestivum*	B	France	16	2009–2010
BgTR_B(8-16)	triticale				
Bgta_C(1)	*Triticum aestivum*	C	Poland	10	2009–2010
BgTR_C(2-10)	triticale				
Bgta_D(1-3)	*Triticum aestivum*	D	Israel	7	1980’s
Bgtd_D(4-6)	*Triticum durum*				
Bgtdic_D(7)	*Triticum diccocoides*				

**Table 2:** **Wheat, triticale and rye cultivars used to characterize the virulence profile of the isolate**s

**Cereal**	**Cultivar**	**Resistance gene**^**a**^	**Breeding company**^**b**^
*Triticum aestivum*	Cerco	None	–
	Axminster	*Pm1a*	–
	Galahad	*Pm2*	–
	Asosan	*Pm3a*	–
	Chul	*Pm3b*	–
	Sonora	*Pm3c*	–
	Broom	*Pm3d*	–
	Khapli	*Pm4a*	–
	Weihenste	*Pm4b*	–
	Hope	*Pm5a*	–
	Ibis	*Pm5b*	–
	Holger	*Pm6*	–
	Kavkaz	*Pm8*	–
	Maris Dove	*Mld*, *Pm2*	–
	Sicco	*Pm5a*, *MlSi2*	–
Triticale	Lamberto	–	Danko, Poland
	Krakowiak	–	Danko, Poland
	Moderato	–	Danko, Poland
	Grenado	–	Danko, Poland
	Maximal	–	Agri-Obtentions, France
	Grandval	–	Agri-Obtentions, France
	Borodine	–	Serasem, France
	Ragtac	–	RAGT, France
	Joyce	–	Sem-Partners, France
	Tribeca	–	Fl. Desprez, France
	Talentro	–	SW Seed, Sweden
	Cultivo	–	SW Seed, Sweden
	Agostino	–	SW Seed, Sweden
	Partout	–	Nordsaat Saatzucht GmbH, Germany
	Amarillo	–	Nordsaat Saatzucht GmbH, Germany
*Secale cereale*	Dankowsky Złote	–	Danko, Poland

^a^Standard wheat differentials with known powdery mildew resistance genes were used.

^b^Fifteen triticale cultivars and one rye cultivar from different European breeding companies were used.

**Table 3:** **General reaction responses of*Blumeria graminis*isolates collected from different hosts on triticale, wheat and rye cultivars**^**a**^

**Source hosts (origin of isolates)**^**b**^	**Inoculated hosts**		
	**Triticale**	**Wheat**	**Rye**
*B.g.* f.sp. ‘triticale’ (*n* = 35)	+++	++	−
*B.g.* f.sp. *tritici* (*n* = 35)	+	+++	−
*B.g.* f.sp. *secalis* (*n* = 2)	+	−	+++

^a^ + indicates infection, − indicates no infection; a higher amount of + corresponds to a higher aggressiveness. Aggressiveness is interpreted here both as a quantitative and qualitative component of pathogenicity. Quantitatively as a higher amount of colonies with well developed hyphae and abundant conidia. Qualitatively as more isolates which are able to infect a particular host or cultivar.

^b^ Number in parenthesis indicates the number of isolates from each source host that were used in this experiment.

### Virulence structure

**Figure 1:**
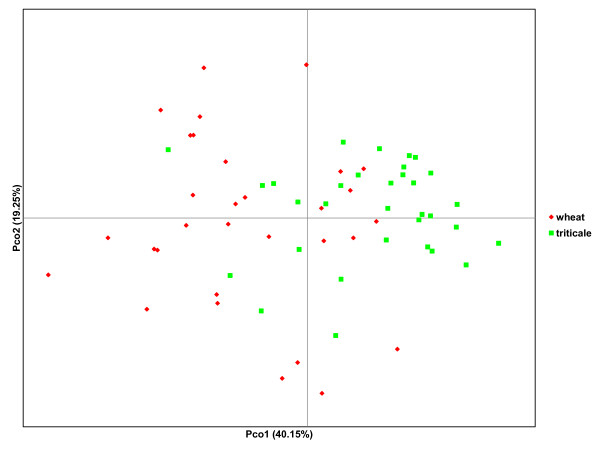
**Principal coordinate graph (PCA) based on the virulence difference matrix on the inoculated wheat lines.** Isolates were collected from triticale and wheat and sampled in four different regions.

The virulence structure analysis comprised isolates collected from triticale (35) and wheat (35) (Table 1), representing the recent expansion of the host range of *B.g.* f.sp. *tritici* in Europe. Among the 70 *B. graminis* isolates, there were 63 virulence profiles identified for their virulence on the wheat lines. The principal component analysis (PCA) plot of the virulence differences on the inoculated wheat lines showed some degree of differentiation between the two groups of isolates (Figure 1). Isolates collected from wheat clustered more on the left side of the plot and isolates collected from triticale clustered more on the right side of the plot. The first two axes explain 59.40% of the total variation. The isolates collected from wheat and triticale differentiated significantly in their virulence profile, with an overall PhiPT value of 0.160 (*P* < 0.01). However, only 16% of the total variation is attributable to the differentiation among populations. Pairwise PhiPT estimates of the virulence profiles on the wheat lines among the *B. graminis* isolates sampled in four different regions are presented in Table 4. Only the virulence profiles from the old isolates sampled in Israel in the 1980’s differed significantly from the virulence profiles of the isolates sampled in Belgium, France and Poland in 2009–2010. The virulence profiles of the isolates sampled in Belgium, France and Poland did not differ significantly.

**Table 4:** **Pairwise PhiPT estimates of the virulence profiles on wheat and triticale cultivars among the*Blumeria graminis*isolates sampled in different regions**

**Virulence profiles on the wheat lines**				
Belgium	France	Poland	Israel	
Belgium	0			
France	0.000^ns^	0		
Poland	0.063^ns^	0.031^ns^	0	
Israel	0.250^**^	0.371^**^	0.577^**^	0
**Virulence profiles on the triticale cultivars**				
Belgium	France	Poland		
Belgium	0			
France	0.000^ns^	0		
Poland	0.016^ns^	0.000^ns^	0	

Negative PhiPT estimates were converted to zero.

*P*-values were estimated after 999 permutations; ^ns^*P* > 0.01; ^**^P < 0.001.

Of the 35 *B.g.* f.sp. *tritici* isolates, only 5 isolates, sampled in the North and Centre of France, were pathogenic on triticale. The remaining 30 *B.g.* f.sp. *tritici* isolates formed one virulence profile, unable to infect triticale. Therefore, only the isolates which were pathogenic on triticale were included in the analysis. Among the 40 isolates pathogenic on triticale (5 collected from wheat and 35 collected from triticale), 14 unique virulence profiles were found. In general, all 40 isolates were highly pathogenic on most of the triticale cultivars. If isolates collected from wheat were pathogenic on triticale, their virulence profile did not differ significantly from the virulence profiles of the isolates collected from triticale, with an overall PhiPT value of 0.021 (*P* > 0.01). Pairwise PhiPT estimates of the virulence profiles among the *B. graminis* isolates pathogenic on triticale sampled in Belgium, France and Poland (Table 4) indicated that their virulence profiles did not differ significantly among regions.

### Phylogeny

**Figure 2:**
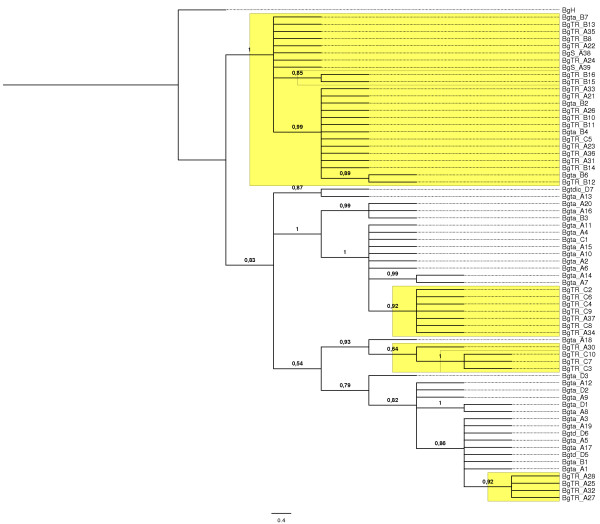
**Phylogeny inferred by Bayesian analysis from the combined* tub2 *and* EF1α *sequence loci.** The 50% majority rule consensus tree is shown. Maximum likelihood analysis recovered the same topology. The numbers below branches are Bayesian posterior probabilities. Branch lengths are proportional to the number of steps (character changes) along the branch. Labels on the phylogeny are isolates collected from different hosts at different locations: BgTR triticale, Bgta wheat, Bgtd durum wheat, Bgtdic wild emmer wheat, BgS rye, BgH Barley; A Belgium, B France, C Poland and D Israel. Yellow shaded areas represent isolates pathogenic on triticale.

A total of 3546 nucleotides from four nuclear gene regions for all isolates were sequenced: the internal transcribed spacer region of nuclear rDNA (ITS), chitin synthase (*chs1*), β-tubulin (*tub2*) and translation elongation factor 1-α (*EF1α*). Summaries of the phylogenetic information for the four loci are shown in Table 5. An isolate from barley (*B.g.* f.sp. *hordei*) retrieved from GenBank was included as a reference. There were no parsimony informative characters for ITS and *chs1*, so for these nuclear gene regions isolates collected from triticale, wheat and rye grouped in the same clade. Therefore, these two regions were excluded from further analysis. The combined datasets of *tub2* and *EF1α* accounted for 299 variable characters of which 41 were parsimony informative. The phylogeny of these combined datasets inferred by Bayesian analysis is presented in Figure 2. The same topology resulted from the maximum likelihood analysis. The 50% majority rule consensus tree shows that the isolates are separated into two major clades, supported by high posterior probabilities. One clade consists only of isolates pathogenic on triticale, which includes the isolates collected from wheat but pathogenic on triticale and the 2 isolates collected from rye (also pathogenic on 2 out of 15 triticale cultivars). This clade is distinct from another clade which consists of all isolates collected from wheat, but non-pathogenic on triticale, as well as of some isolates collected from triticale. All other branches or clades indicated on the phylogenetic tree were well supported by high Bayesian posterior probabilities.

**Table 5:** Phylogenetic information of the nuclear regions used in this study for the individual and combined datasets

	**DNA sequence region**				
	**ITS**	***chs1***	***tub2***	***EF1α***^**c**^	**Combined**^**d**^
isolates^a^	73	73	73	69	69
haplotypes	2	2	8	17	29
sites	483	473	1425	1183	2608
constant characters	472	420	1329	980	2309
variable characters (uninformative)	11	53	92	166	258
parsimony informative characters	0	0	4	37	41
% informative characters	0	0	0.28	3.13	1.57
Model AIC^b^	K80	GTR	GTR	GTR + G	GTR + G

^a^Isolates were collected from triticale (35), wheat (35) and rye (2). We also included an isolate from barley (*B.g.* f.sp. *hordei*) in our analysis for comparison. These sequences were retrieved from GenBank with accession numbers AJ313137, AB273568, AJ313149 and HM538424.1, respectively for each locus.

^b^Model of nucleotide substitution [49,50].

^c^We were unable to obtain *EF1α* sequences for the isolates BgTR_A29, Bgta_B5, BgTR_B9 and Bgtd_D4.

^d^Only the loci *tub2* and *EF1α* were included in the combined dataset.

For the total population of 69 isolates, 29 haplotypes were identified for the combined data of loci *tub2* and *EF1α* (Table 5). The isolates which were pathogenic on triticale displayed 10 of those haplotypes, while the isolates non-pathogenic on triticale were divided into 17 haplotypes in the combined data tree.

### Phylogeography

**Figure 3:**
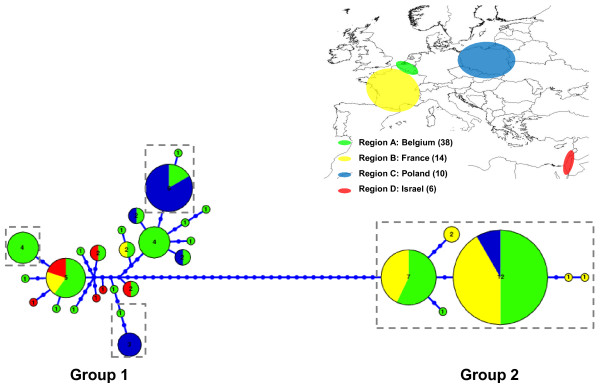
**Multilocus haplotype network from the 50% majority rule consensus tree inferred by Bayesian analysis.** The network was performed in the program Haploviewer. The *B.g.* f.sp. *hordei* haplotype inferred from GenBank was excluded out of this analysis because of its large mutational distance with the isolates from this study. The circle area of each haplotype, coded as a number, is proportional to its frequency. Dots represent inferred unsampled or extinct haplotypes. Haplotypes framed with a dotted line represent isolates pathogenic on triticale.

To determine phylogeographical relationships among isolates, a multilocus haplotype network was constructed combining *tub2* and *EF1α* (Figure 3). The *B. graminis* f.sp. *hordei* haplotype inferred from GenBank was excluded from this analysis because of its large mutational distance with the isolates from this study. Among 69 isolates, 29 haplotypes were recognized. The haplotypes clustered into two groups (1 and 2), corresponding to the clades in the phylogenetic tree (Figure 2), separated by a minimum of 33 fixed differences (Figure 3). This suggests that many possible haplotypes were not sampled or they are extinct haplotypes. All isolates pathogenic on triticale displayed different haplotypes than the isolates non-pathogenic on triticale. Although group 2 consists only of isolates pathogenic on triticale, some of the haplotypes from isolates pathogenic on triticale were also distributed at different places in group 1 of the network. Moreover, these haplotypes clustered at or near the tips of the network. The majority of unique haplotypes are represented by isolates non-pathogenic on triticale, demonstrating that these isolates are more diverse than the isolates pathogenic on triticale.

The haplotype network also revealed some pattern of geographical association among the *B. graminis* haplotypes. All isolates sampled in France, which were pathogenic on triticale, belong to group 2. All isolates sampled in Poland except one belong to group 1. The isolates sampled in Belgium formed haplotypes distributed over both groups. The old isolates from the Middle East formed diverse haplotypes, mostly derived near the centre of the multilocus network. This underscores that this region is the centre of diversity.

### Population structure

Differentiation was estimated between populations of *B. graminis* divided by geographic region, host origin and pathogenicity (Table 6). Geographic differentiation was detected when isolates from all hosts were included in the analysis, as well as among isolates sampled on triticale, despite of the small sample sizes. More particularly, within the population of isolates sampled on triticale, significant differentiation was found among the isolates collected in the different regions, except between the isolates collected in region A (Belgium) and region B (France). On the other hand, no significant differentiation was found between the old wheat isolates from Israel and the wheat isolates collected in 2009–2010 in different regions in Europe. Significant differentiation was detected between populations pathogenic and non-pathogenic on triticale. This significance was also reflected when the isolates were divided by host origin.

**Table 6:** **Population structure of*Blumeria graminis*by geographic region, host origin and pathogenicity**

**Populations compared**^**a**^	**S**_**nnb**_	**H**_**STc**_
By geographic region		
Region A (36) vs. region B (14) vs. region C (10) vs. region D (6)	0.5468^*^	0.0469^**^
By host origin		
triticale (33) vs. wheat (33)	0.8654^**^	0.0397^**^
By year of collection within host origin wheat		
Region D 1980’s (6) vs. Region A-B-C 2009–2010	0.7283^ns^	−0.0025^ns^
By geographic region within host origin triticale		
region A (16) vs. region B (8) vs. region C (9)	0.5899^**^	0.1008^**^
region A (16) vs. region B (8)	0.6597^ns^	0.0019^ns^
region A (16) vs. region C (9)	0.7667^*^	0.1116^*^
region B (8) vs. region C (9)	0.8677^*^	0.1428^*^
By pathogenicity		
Pathogenic (37) vs. non-pathogenic (29) on triticale	0.9811^**^	0.0565^**^

Region A represents Belgium, region B France, region C Poland and region D Israel.

^a^Sample sizes are in parentheses.

^b^S_nn_ (the nearest neighbor statistic) measures how often the most similar sequence (or sequences) is from the same designated population. S_nn_ has a high power with small population sizes.

^c^H_ST_ is a measure of population subdivision that estimates F_ST_ among populations.

*P*-values were estimated after 1000 permutations; ^ns^*P* > 0.01; ^*^P < 0.01; ^**^P < 0.001.

## Discussion

### Host specificity

Recent research has provided a compelling amount of evidence that powdery mildew on triticale has emerged most probably through a host range expansion of powdery mildew on wheat [16]. To properly infer the evolutionary processes that led to this host range expansion, powdery mildew populations both from the present (wheat) and new hosts (triticale) were sampled. This study represents a successful attempt to establish a link between virulence profile data from pathogenicity tests and (phylo)genetic data.

Giraud et al. [24] defined host range expansions as the evolution of the ability to exploit a novel host in addition to the host of origin. The pathogenicity tests show that *B. graminis* isolates collected from triticale are highly pathogenic on most of the tested triticale cultivars. Moreover, these isolates were also able to infect several wheat cultivars (their previous hosts), although some variation in aggressiveness compared to isolates collected from wheat was observed. Among the 70 isolates used in this study, 63 virulence profiles were identified on the wheat lines with known powdery mildew resistance genes. On the other hand, among the 40 isolates pathogenic on triticale, only 14 displayed unique virulence profiles on the triticale cultivars. In general, all virulence profiles identified on the triticale cultivars were highly aggressive. Altogether, these results suggest that the disease resistance in triticale has largely been based on major genes which follow the gene-for-gene response [5]. This disease resistance has probably been overcome by a few modifications in the effector repertoire of *B. graminis* f.sp. *tritici* to adapt to the closely related triticales genotypes. The fact that it has taken decades for the resistance of triticale to be overcome is probably because the total acreage of the crop is still small compared to wheat and barley. Additionally, triticale genotypes have probably more than one resistance gene against wheat-adapted isolates of the fungus. A similar breakdown of disease resistance was found for *Magnaporthe oryzae* f.sp. *oryzae*, which likely emerged from a host range expansion from a Setaria millet, possibly brought about by the loss of a strain-specific effector gene, which allowed the fungus to colonize rice [22,25,26]. Other recent examples of host range expansions of pathogens to triticale are the spreading of rice blast disease to wheat and triticale in Brazil [27] and the spreading of scald (*Rhynchosporium secalis*) of rye to triticale [28]. The pathogenicity tests, showing that isolates collected from wheat and rye were occasionally able to infect triticale, corroborates previous findings of Linde-Laursen [29] and Walker et al. [16], and underscores the close relatedness of this intergeneric hybrid with its parents wheat and rye.

Pursuing a phylogenetic approach, we tried to develop a coherent view on the genetic diversity of powdery mildew isolates. No genetic diversity was observed for ITS region and *chs1* gene due to the closely related hosts. However, the combined datasets of *tub2* and *EF1α* accounted for 299 variable characters (of 2608 in total) of which 41 were parsimony informative. Bayesian posterior probability values identified two statistically significant clades, which to a certain extent correlated with pathogenicity. One clade consisted only of isolates pathogenic on triticale, distinct from another clade which consisted mostly of isolates non-pathogenic on triticale. A higher number of haplotypes was observed for the isolates which were non-pathogenic on triticale, compared to the isolates pathogenic on triticale. These results support the second hypothesis that genetic diversity is highest in the wheat powdery mildew populations compared to the recently emerged triticale powdery mildew populations. It was expected that the genetic diversity would be greatest in the powdery mildew population on wheat compared to the recently emerged population on triticale, because of their longer-standing accumulation of mutations.

Although the phylogenetic analysis did not cluster all isolates pathogenic on triticale in the same clade, it is highly likely that there is population differentiation according to host specificity. This is supported by the significant measures of S_nn_ and H_ST_ when populations pathogenic and non-pathogenic on triticale were compared. Additionally, the haplotype network, resulting in two groups (1 and 2), separated by a minimum of 33 fixed differences, highlighted some degree of genetic isolation. This host range expansion may eventually result in a host shift speciation, which means that a subset of the population speciates on triticale, thereby becoming incapable of infecting wheat, with cessation of gene flow from the population infecting wheat [24]. Historical cases of fungal disease emergence in cereals due to ecological speciation by host shifts are the emergence of *Mycosphaerella graminicola*[30] and *Rhynchosporium secalis*[28] on different cereal hosts.

### Geographic subdivision

Part of the first hypothesis in this study was that the triticale powdery mildew population is structured by geography. To validate this hypothesis, powdery mildew isolates at different triticale breeding areas in Europe were sampled.

Regarding the virulence profiles on the wheat lines, results demonstrated that the old isolates sampled in Israel in the 1980’s differed significantly from the isolates sampled in Belgium, France and Poland in 2009–2010. A possible explanation for this finding is that in a period of 30 years, host populations have changed, more particularly, they carry different *R* genes and therefore isolates with other *AVR*-like genes are now more adapted and thus more virulent. Eshed and Wahl [31] described that isolates from Israel have a wider host range than those from elsewhere in the world, reflecting the broader genetic diversity of host plants in the Middle East. However, this could not be confirmed in the present study, as the isolates from Israel were only pathogenic on wheat, and not on triticale or rye. If isolates were pathogenic on triticale, their virulence profiles on the triticale cultivars did not differ significantly among the different sampled regions (Belgium, France and Poland). Probably the triticale cultivars tested in this study, although from different European breeding companies, have the same narrow genetic background carrying the same *R* genes for powdery mildew resistance [16] and therefore no differences in virulence profiles were found among regions. In surveys of wheat powdery mildew, it is indeed described that virulence frequencies are highly influenced by the resistance genes carried by cultivars grown in a particular area [32,33].

The multilocus haplotype network demonstrated that haplotypes of isolates pathogenic on triticale are distributed at different sites in the network, but always clustered at or near the tips of the network. Therefore, it is likely that the host range expansion of wheat isolates to the new host triticale occurred recently and multiple times at different locations in Europe. The haplotype network also revealed some pattern of geographical association among the *B. graminis* haplotypes, and this finding was also supported by significant measures of S_nn_ and H_ST_ when populations among geographic regions were compared. An exception to this rule are the isolates sampled in Belgium and France for which no significant differentiation was found. It can be envisaged that the small geographical distance between both groups causes this lack of differentiation. Population genetic analyses of wheat powdery mildew and grape powdery mildew also demonstrated that populations were geographically differentiated [34,35]. The network might also give evidence for long distance spore dispersal as haplotypes (pathogenic on triticale) from Belgium and Poland were distributed over both groups of the network. Long-distance dispersal of fungal spores by the wind can spread plant diseases across and even between continents, which holds true for powdery mildew [36,37]. The old isolates from the Middle East formed diverse haplotypes, mostly derived near the centre of the multilocus network. This might suggest that this region is representing the ancestral population and the centre of diversity of *B. graminis*, which was already stated by Wyand and Brown [14].

## Conclusions

The present study pursued a combined pathological and (phylo)genetic approach to explain the evolutionary processes that led to the host range expansion of powdery mildew from wheat to triticale. To our knowledge, this is the first population study of powdery mildew on its new host triticale. Based on the genomic regions of *tub2* and *EF1α*, the distribution of genetic variation demonstrated to be geography dependent. In addition, evidence is brought forward showing that the host range expansion of wheat isolates to the new host triticale occurred recently and multiple times at different locations in Europe. Finally, this study reveals a genetic structure in *Blumeria graminis*, which is to a certain extent correlated with pathogenicity. Two genetically distinct groups could be detected of which one group consisted exclusively of isolates pathogenic on triticale. Other genomic regions, including *AVR* effector-like genes, should be investigated to support this genetic differentiation.

## Methods

### Fungal isolates

A total of 72 isolates were obtained from triticale (35), *Triticum aestivum* (31), *T. durum* (3), *T. diccocoides* (1) and *Secale cereal*e (2) (Table 1). The surveyed area covered three triticale and wheat breeding regions in Europe. Region A represents Belgium, Region B France and Region C Poland. Isolates were derived from infected leaves collected in different fields during 2009 and 2010. All isolates were made single colony and cultured on detached leaves on water agar (5 g L^−1^) amended with benzimidazole (40 mg L^−1^) in petri dishes [38]. Cultures were maintained at 18°C with a 16 h photoperiod and transferred to new leaves every 14 days. The following susceptible host cultivars were used for the maintenance of the different *formae speciales*: Cerco (wheat), Lamberto (triticale) and Dankowsky Złote (rye). Old isolates from Israel were included, which is region D in this study (Table 1). These isolates were sampled across Israel in the 1980’s from three *Triticum* spp. (*T. aestivumT. durum* and *T. diccocoides*) by N. Eshed and A. Dinoor (Hebrew University of Jerusalem, Rehovot, Israel). Detailed information about the isolates used in this study are listed in Additional file 3.

### Plant material and inoculation

The virulence of each isolate was assessed on a set of 15 wheat differential lines (Table 2) with known powdery mildew resistance genes (*Pm*) in order to determine the virulence genes associated with each isolate. These differential lines were kindly provided by James Brown from the John Innes centre and cover the most common resistance genes used in European wheat breeding [39]. We also determined a virulence profile on 15 commercial triticale cultivars and 1 rye cultivar (Table 2) from different European breeding companies.

Inoculation was performed in a settling tower measuring 300 mm high and 103 mm diameter by uniformly dispersing conidia [38]. Inoculation was carried out on 2-week old seedlings. For each isolate × cultivar combination, a petri dish containing three leaf segments of the cultivar and one leaf segment of a susceptible control was placed at the bottom of the tower, and conidia of the isolate were blown into a upper hole of the tower. Two independent inoculations were done for each isolate × cultivar combination. Inoculum density was ca. 20 conidia mm^−2^. Infection types were scored 12 days after inoculation using a modified 0–4 scale [40,41]. Cultures were maintained on the same medium and under the same conditions as mentioned above. Infection types of isolates of *Blumeria graminis* collected from wheat and triticale in different regions in Europe on wheat and triticale cultivars are available in Additional files 1 and 2, respectively.

### Virulence structure analysis

The 0–4 scale used for infection types was converted into a binary code of 1 (scores 2–4) and 0 (scores 0–1) that corresponded to virulence or avirulence of the isolate to a cultivar, respectively. In this way, a virulence profile for each isolate was defined on the respective wheat and triticale cultivars. A matrix of virulence differences between isolates collected from triticale and wheat was derived with GenAlEx 6 [42]. This matrix was plotted in a two-dimensional principal coordinate graph. The level of differentiation for virulence profiles between the *B. graminis* populations on wheat and triticale was computed in GenAlEx 6 with PhiPT with 999 permutations of the dataset. PhiPT is an F_ST_ analogue, which represents the proportion of variance among populations, relative to the total variance.

### DNA extraction, PCR and sequencing

For DNA extraction, conidia were collected by shaking contaminated leaves into a plastic tube. DNA was extracted with the DNeasy Plant Mini Kit (Qiagen), used according to manufacturer’s instructions, except that the conidia were lyophilized and ground before DNA extraction. Four nuclear loci were PCR-amplified and sequenced from each isolate, using published primers: ITS region [43], chitin synthase *chs1*[15], β-tubulin *tub2*[14] and elongation factor *EF1α*[16]. The PCR reactions were carried out in a total volume of 50 μL. Reaction components included 5 μL of 10× PCR buffer (Applied Biosystems), 2.5 μL dNTPs (5 mM each), 5 μL each of two primers (5 μM), 25 mM MgCl_2_, 1 U *Taq* DNA polymerase (Applied Biosystems) and 1 μL DNA template. Cycling conditions included an initial denaturation at 94°C for 5 min followed by 35 cycles with a denaturation step at 94°C for 45 s, annealing at 50–61°C for 30 s [16], extension at 72°C for 1 min, followed by a final extension at 72°C for 5 min. PCR product were purified with Qiaquick spin columns (Qiagen). Fragments of the appropriate size were sequenced in both directions by the commercial Macrogen facility (Macrogen, Seoul, Korea). Sequences of *tub2* and *EF1α* of all isolates used in this study are deposited in GenBank and the accession numbers are available in Additional file 3.

### Phylogenetic analysis and haplotype network construction

Sequences were aligned using CLUSTALW [44,45] and manually refined in BioEdit 7.0.9 [46]. Consensus sequences were compared with those published for reference isolates of *B. graminis* from the GenBank database. The phylogenetic analyses were conducted using maximum likelihood (ML) and Bayesian methods. For ML, the software PAUP* 4.0b10 was used [47]. The Bayesian analysis was carried out using MrBayes 3.1.2 [48].

The Akaike information criterion (AIC) as implemented by MODELTEST 3.7 [49,50] was used to determine the DNA bases substitution model more fit to the data (Table 5). Two independent analyses were run in MrBayes starting with a random tree. Markov chains were run for 10^6^ generations, burn-in values were set for 10^4^ generations, and trees were sampled every 100 generations. The remaining trees were used to calculate a 50% majority rule consensus tree and to determine the posterior probabilities.

A multilocus haplotype network was generated using phylogenetic algorithms with migration [51]. The 50% majority rule consensus tree inferred by Bayesian analysis was used to estimate a haplotype network performed in the program Haploviewer [51].

### Population structure analysis

Differentiation among geographic regions, host origin and pathogenicity was estimated on combined multilocus sequences of *tub2* and *EF1α*. According to Brewer et al. [35], the nearest neighbor statistic (S_nn_) measures were selected for analysis because it has high power with small population sizes. This statistic measures how often the most similar sequence (or sequences) is from the same designated population. Additionally, H_ST_ was estimated, a measure of population subdivision that estimates F_ST_ among haplotypes. Both S_nn_ and H_ST_ were calculated using the program DnaSP v5 [52] and *P*-values were estimated by permutation tests (1000 replications).

## Abbreviations

AIC, Akaike information criterion; AVR, avirulence; B.g., Blumeria graminis; BgH, Blumeria graminis from Hordeum; BgS, Blumeria graminis from Secale; Bgta, Blumeria graminis from Triticum aestivum; Bgtd, Blumeria graminis from Triticum durum; Bgtdic, Blumeria graminis from triticum diccocoides; BgTR, Blumeria graminis from Triticale; chs, chitin synthase; EF, Elongation factor; f.sp, formae speciales; Fst, Fixation index; Hst, Haplotype statistic; ITS, internal transcribed spacer; ML, maximum likelihood; nsS, not significant; PCA, principle component analysis; PhiPT, portion of variance among populations relative to total variance; Pm, Powdery mildew; Snn, nearest neighbor statistic; tub, tubulin.

## Authors’ contributions

KA, MH and GH designed the study. VT conducted the research, analyzed the data, interpreted the results and wrote the manuscript. BB carried out the molecular work. KA, MH and GH assisted with interpreting the results and critically revised the drafts of the manuscript. All authors read and approved the final manuscript.

## Supplementary Material

Additional file 1 Infection types of isolates of *Blumeria graminis* collected from wheat and triticale in different regions in Europe on wheat differential cultivars with known powdery mildew resistance genes. Click here for file

Additional file 2 Infection types of isolates of *Blumeria graminis* collected from wheat and triticale in different regions in Europe on triticale cultivars. Click here for file

Additional file 3 Isolates of *Blumeria graminis* included in this study. This additional file includes detailed information of the isolates used in this study; source host, isolate code, sampling location, year of collection and GenBank accession numbers are given when available. Click here for file
