# Supplementary material for: Phylogeography and virulence structure of the powdery mildew population on its 'new' host triticale
Source: BMC Evol Biol. 2012 Jun 1;12:76. doi: 10.1186/1471-2148-12-76 (PMC3457899; doi:10.1186/1471-2148-12-76)
Supplement: Additional file 1 — Infection types of isolates of Blumeria graminis collected from wheat and triticale in different regions in Europe on wheat differential cultivars with known powdery mildew resistance genes. [file 1471-2148-12-76-S1.pdf]

Additional files

Additional file 1 – Infection types of isolates of *Blumeria graminis* collected from wheat and triticale in different regions in Europe on wheat differential cultivars with known powdery mildew resistance genes

Isolate code[a]	Infection types[b] of *Blumeria graminis* isolates on wheat differential cultivars														
	Cerco (None)	Axminster (*Pm1a*)	Galahad (*Pm2*)	Asosan (*Pm3a*)	Chul (*Pm3b*)	Sonora (*Pm3c*)	Broom (*Pm3d*)	Khapli (*Pm4a*)	Weihenste (*Pm4b*)	Hope (*Pm5a*)	Ibis (*Pm5b*)	Holger (*Pm6*)	Kavkaz (*Pm8*)	Maris Dove (*Mld*, *Pm2*)	Sicco (*Pm5a*, *MlSi2*)
Bgta_A1	4	1	4	0	0	4	4	0	4	3	4	4	4	4	0
Bgta_A2	4	1	4	0	4	4	4	3	0	1	0	4	3	1	1
Bgta_A3	4	0	4	0	0	2	0	0	0	2	2	2	0	1	0
Bgta_A4	4	0	4	4	2	3	4	4	1	2	4	4	2	4	1
Bgta_A5	3	2	4	4	0	3	0	0	0	3	4	2	4	1	0
Bgta_A6	4	2	4	0	1	3	4	0	3	1	3	4	4	3	2
Bgta_A7	4	2	4	3	0	2	4	3	0	4	1	3	1	0	4
Bgta_A8	4	4	4	3	0	0	4	4	1	2	3	4	4	1	0
Bgta_A9	4	4	4	0	0	0	0	0	0	3	1	2	4	0	0
Bgta_A10	4	1	3	4	1	2	1	1	0	4	4	4	4	3	2
Bgta_A11	4	0	4	4	2	3	2	2	4	1	3	4	3	1	0
Bgta_A12	4	0	3	3	0	3	3	4	2	4	3	4	3	0	0
Bgta_A13	4	2	4	1	0	4	0	0	4	2	0	3	4	4	0
Bgta_A14	4	0	4	0	2	1	0	1	0	4	1	2	1	2	1
Bgta_A15	4	3	3	4	2	4	2	0	3	2	4	4	0	3	4

Isolate code[a]	Infection types[b] of *Blumeria graminis* isolates on wheat differential cultivars														
	Cerco (None)	Axminster (*Pm1a*)	Galahad (*Pm2*)	Asosan (*Pm3a*)	Chul (*Pm3b*)	Sonora (*Pm3c*)	Broom (*Pm3d*)	Khapli (*Pm4a*)	Weihenste (*Pm4b*)	Hope (*Pm5a*)	Ibis (*Pm5b*)	Holger (*Pm6*)	Kavkaz (*Pm8*)	Maris Dove (*Mld*, *Pm2*)	Sicco (*Pm5a*, *MlSi2*)
Bgta_A16	4	1	4	3	1	0	0	0	0	4	2	4	3	3	3
Bgta_A17	4	1	4	3	0	0	1	1	1	1	4	4	4	1	1
Bgta_A18	4	0	4	0	0	0	0	0	3	1	1	4	4	4	2
Bgta_A19	3	1	3	4	0	2	0	2	3	4	3	4	3	1	4
Bgta_A20	3	3	3	4	2	4	2	1	4	4	4	4	3	1	3
BgTR_A21	4	1	3	0	0	1	0	0	0	0	4	2	4	0	0
BgTR_A22	2	0	0	0	0	1	0	0	0	0	0	0	1	0	0
BgTR_A23	4	0	0	0	0	0	0	0	0	0	2	1	1	0	1
BgTR_A24	3	1	2	0	0	0	0	0	0	0	1	4	4	1	0
BgTR_A25	1	0	1	4	0	0	0	0	0	0	0	4	4	0	0
BgTR_A26	0	0	0	1	0	0	0	0	0	3	0	2	2	0	1
BgTR_A27	3	4	4	4	0	3	0	0	0	2	3	3	4	1	4
BgTR_A28	4	2	2	0	0	0	0	0	0	0	2	4	4	0	2
BgTR_A29	4	1	1	0	0	0	0	0	0	0	0	0	1	0	0
BgTR_A30	3	0	1	3	0	0	0	0	0	1	2	1	2	1	0
BgTR_A31	4	0	4	4	0	0	0	0	0	2	4	2	3	0	0
BgTR_A32	3	0	0	0	0	1	0	0	0	0	2	2	1	1	0
BgTR_A33	4	0	0	1	0	0	0	0	0	2	3	4	2	0	1
BgTR_A34	2	4	0	4	1	4	0	1	0	3	2	2	4	1	2
BgTR_A35	3	0	3	4	0	0	0	0	0	1	2	2	3	0	0

**Isolate code**[a]	**Infection types**[b] **of *Blumeria graminis* isolates on wheat differential cultivars**														
	Cerco (None)	Axminster (*Pm1a*)	Galahad (*Pm2*)	Asosan (*Pm3a*)	Chul (*Pm3b*)	Sonora (*Pm3c*)	Broom (*Pm3d*)	Khapli (*Pm4a*)	Weihenste (*Pm4b*)	Hope (*Pm5a*)	Ibis (*Pm5b*)	Holger (*Pm6*)	Kavkaz (*Pm8*)	Maris Dove (*Mld, Pm2*)	Sicco (*Pm5a, MlSi2*)
BgTR_A36	4	3	0	0	0	0	0	0	0	2	3	3	2	0	3
BgTR_A37	2	0	3	2	0	0	0	0	0	0	1	1	4	0	0
BgS_A38	0	0	0	0	0	0	0	0	0	0	0	0	0	0	0
BgS_A39	0	0	0	0	0	0	0	0	0	0	0	0	0	0	0
Bgta_B1	4	3	3	4	1	2	1	1	2	4	4	3	1	4	2
Bgta_B2	3	2	3	0	0	2	0	0	0	0	0	1	4	0	1
Bgta_B3	4	1	3	4	1	0	2	2	4	4	4	4	4	4	1
Bgta_B4	4	1	0	1	0	3	2	4	0	1	4	1	3	0	1
Bgta_B5	4	1	2	2	0	3	2	3	0	2	4	4	3	3	0
Bgta_B6	3	0	0	0	0	1	0	0	0	0	3	3	3	0	0
Bgta_B7	4	0	0	0	0	0	0	0	0	2	3	3	4	0	0
BgTR_B8	4	0	4	1	0	4	0	0	4	0	1	1	4	1	1
BgTR_B9	2	0	2	0	0	3	0	1	0	4	0	4	4	1	0
BgTR_B10	4	0	0	0	0	0	1	0	0	0	2	4	4	0	0
BgTR_B11	3	0	0	0	0	1	0	0	0	2	2	4	3	0	0
BgTR_B12	4	0	0	0	0	0	0	0	0	1	2	4	2	0	0
BgTR_B13	4	1	4	4	0	2	0	0	2	2	4	4	4	1	0
BgTR_B14	4	2	4	3	1	1	3	0	0	1	2	4	2	0	0
BgTR_B15	3	1	0	3	0	0	0	0	0	0	2	2	2	1	0
BgTR_B16	3	4	2	4	0	3	3	2	0	3	1	2	3	2	1

Isolate code[a]	Infection types[b] of *Blumeria graminis* isolates on wheat differential cultivars														
	Cerco (None)	Axminster (*Pm1a*)	Galahad (*Pm2*)	Asosan (*Pm3a*)	Chul (*Pm3b*)	Sonora (*Pm3c*)	Broom (*Pm3d*)	Khapli (*Pm4a*)	Weihenste (*Pm4b*)	Hope (*Pm5a*)	Ibis (*Pm5b*)	Holger (*Pm6*)	Kavkaz (*Pm8*)	Maris Dove (*Mld*, *Pm2*)	Sicco (*Pm5a*, *MlSi2*)
Bgta_C1	4	0	4	0	0	4	0	0	0	3	4	3	4	1	0
BgTR_C2	2	2	0	0	0	0	0	0	0	0	0	2	4	0	1
BgTR_C3	4	0	4	0	0	3	0	0	1	1	0	1	4	0	0
BgTR_C4	4	0	2	0	0	1	1	0	0	0	0	0	1	0	0
BgTR_C5	1	1	0	4	0	4	2	1	0	1	0	3	4	0	0
BgTR_C6	2	1	0	4	0	1	0	1	0	0	0	3	3	1	0
BgTR_C7	4	2	3	4	0	3	0	1	0	1	3	2	1	0	0
BgTR_C8	4	0	0	0	0	0	0	0	1	1	3	1	4	0	0
BgTR_C9	4	2	4	4	0	4	1	3	0	1	4	4	4	1	0
BgTR_C10	4	1	0	0	2	3	0	1	0	0	1	3	2	0	0
Bgta_D1	4	3	1	0	0	3	0	0	0	2	2	0	0	0	3
Bgta_D2	4	4	0	1	0	2	2	0	4	2	4	2	3	0	3
Bgta_D3	4	4	0	0	0	0	4	1	0	4	4	4	0	0	4
Bgtd_D4	3	4	0	0	0	0	0	0	0	2	4	0	1	0	1
Bgtd_D5	4	2	0	3	0	2	0	0	0	2	2	0	0	0	4
Bgtd_D6	4	4	4	4	4	3	4	0	0	2	4	3	2	1	4
Bgtdic_D7	4	4	0	2	1	3	2	0	0	4	4	4	4	0	4

[a]Isolate codes represent isolates collected from different hosts at different locations: BgTR triticale, Bgta wheat, Bgtd durum wheat, Bgtdic wild emmer wheat, BgS rye; A Belgium, B France, C Poland and D Israel. Detailed information about the sampling location and year of collection are shown in additional file 3.

[b]The 0-4 scale [40,41] for infection types was converted into a binary code of 1 (scores 2-4) and 0 (scores 0-1) that corresponded to virulence or avirulence of the isolate to a cultivar, respectively.
