# Supplementary material for: Phylogeography and virulence structure of the powdery mildew population on its 'new' host triticale
Source: BMC Evol Biol. 2012 Jun 1;12:76. doi: 10.1186/1471-2148-12-76 (PMC3457899; doi:10.1186/1471-2148-12-76)
Supplement: Additional file 2 — Infection types of isolates of Blumeria graminis collected from wheat and triticale in different regions in Europe on triticale cultivars. [file 1471-2148-12-76-S2.pdf]

Additional files

Additional file 2 – Infection types of isolates of *Blumeria graminis* collected from wheat and triticale in different regions in Europe on triticale cultivars

Isolate code[a]	Infection types[b] of *Blumeria graminis* isolates on triticale cultivars														
	Lamberto	Krakowiak	Moderato	Grenado	Maximal	Grandval	Borodine	Ragtac	Joyce	Tribeca	Talentro	Cultivo	Agostino	Partout	Amarillo
BgTR_A21	4	4	1	0	4	4	3	4	4	4	4	0	4	4	3
BgTR_A22	4	4	4	0	4	3	4	2	4	4	4	0	3	1	4
BgTR_A23	4	4	4	0	4	4	4	4	3	3	3	0	3	1	3
BgTR_A24	2	4	1	0	3	4	1	3	4	3	4	0	2	2	3
BgTR_A25	4	4	1	0	4	4	4	3	4	4	4	0	4	3	0
BgTR_A26	3	4	4	0	3	4	4	4	4	4	4	0	4	4	3
BgTR_A27	4	4	3	0	4	4	4	4	4	4	4	0	4	1	4
BgTR_A28	3	4	4	0	4	4	4	4	4	4	3	0	4	0	4
BgTR_A29	4	4	4	0	4	4	2	1	3	2	2	0	3	0	4
BgTR_A30	4	3	0	0	4	4	3	4	4	3	4	0	3	4	4
BgTR_A31	4	4	3	0	4	4	4	4	4	4	4	0	4	4	3
BgTR_A32	3	4	1	0	3	3	3	4	4	3	4	0	4	3	3
BgTR_A33	4	4	4	0	4	4	4	3	4	4	4	0	4	4	0
BgTR_A34	4	4	4	0	4	4	4	3	4	4	4	0	4	4	3
BgTR_A35	4	4	2	0	4	4	3	4	4	4	4	0	4	2	3

Isolate code[a]	Infection types[b] of *Blumeria graminis* isolates on triticale cultivars														
	Lamberto	Krakowiak	Moderato	Grenado	Maximal	Grandval	Borodine	Ragtac	Joyce	Tribeca	Talentro	Cultivo	Agostino	Partout	Amarillo
BgTR_A36	4	4	3	0	4	4	4	4	4	4	3	0	4	2	1
BgTR_A37	2	3	0	0	1	4	1	4	3	3	4	0	2	0	1
BgS_A38	0	0	0	0	1	2	0	0	0	2	0	0	0	0	0
BgS_A39	0	1	0	0	0	2	0	0	0	2	0	0	0	0	0
Bgta_B2	3	1	4	0	3	4	4	4	4	4	1	0	3	1	4
Bgta_B4	4	4	1	0	4	3	4	4	4	4	4	0	4	1	3
Bgta_B5	4	4	4	0	4	4	4	4	4	4	3	0	4	4	4
Bgta_B6	3	4	3	0	4	3	4	4	4	4	4	0	4	4	4
Bgta_B7	4	4	2	0	4	4	3	4	4	4	4	0	4	1	4
BgTR_B8	2	4	0	0	4	3	1	0	0	4	0	0	2	4	3
BgTR_B9	4	4	1	0	4	4	4	3	4	4	4	0	4	4	4
BgTR_B10	3	3	2	0	4	4	4	4	4	4	3	0	4	1	1
BgTR_B11	4	4	3	0	4	4	4	3	4	3	4	0	4	3	1
BgTR_B12	3	4	3	0	4	4	4	4	4	4	4	0	4	4	3
BgTR_B13	4	4	3	0	4	4	2	1	2	4	1	0	3	3	3
BgTR_B14	4	4	1	0	4	3	4	4	3	4	3	0	3	4	2
BgTR_B15	4	4	2	0	4	4	4	4	4	4	4	0	3	2	1
BgTR_B16	4	4	1	0	4	4	3	4	4	4	3	0	4	4	4
BgTR_C2	3	4	0	0	4	3	2	4	4	3	3	0	4	3	3
BgTR_C3	4	4	4	0	4	4	0	0	0	4	0	0	0	1	4

Isolate code[a]	Infection types[b] of *Blumeria graminis* isolates on triticale cultivars														
	Lamberto	Krakowiak	Moderato	Grenado	Maximal	Grandval	Borodine	Ragtac	Joyce	Tribeca	Talentro	Cultivo	Agostino	Partout	Amarillo
BgTR_C4	4	4	3	0	4	1	4	3	3	3	4	0	4	1	4
BgTR_C5	4	4	1	0	4	3	4	3	3	4	4	0	4	3	2
BgTR_C6	4	4	2	0	4	3	4	3	4	4	3	0	4	3	0
BgTR_C7	4	4	4	0	4	4	3	4	4	3	4	4	4	2	1
BgTR_C8	4	4	4	1	4	4	3	4	3	2	4	4	4	1	2
BgTR_C9	3	4	4	0	4	4	4	4	4	3	4	0	4	4	1
BgTR_C10	4	3	3	0	4	4	1	4	4	2	3	3	3	3	1

[a]Isolate codes represent isolates collected from different hosts at different locations: BgTR triticale, Bgta wheat, BgS rye; A Belgium, B France, and C Poland. Detailed information about the sampling location and year of collection are shown in additional file 3. Only isolates pathogenic on triticale are presented, the remaining isolates resulted in a score 0 on all triticale cultivars.

[b]The 0-4 scale [40,41] for infection types was converted into a binary code of 1 (scores 2-4) and 0 (scores 0-1) that corresponded to virulence or avirulence of the isolate to a cultivar, respectively.
