# Supplementary material for: Phylogeography and virulence structure of the powdery mildew population on its 'new' host triticale
Source: BMC Evol Biol. 2012 Jun 1;12:76. doi: 10.1186/1471-2148-12-76 (PMC3457899; doi:10.1186/1471-2148-12-76)
Supplement: Additional file 3 — Isolates of Blumeria graminis included in this study. This additional file includes detailed information of the isolates used in this study; source host, isolate code, sampling location, year of collection and GenBank accession numbers are given when available. [file 1471-2148-12-76-S3.pdf]

Additional files

Additional file 3 – Isolates of *Blumeria graminis* included in this study

Source host, isolate code, sampling location, year of collection and GenBank accession numbers are given when available.

Source host[a]	Isolate code	Sampling location	Year of collection	*tub2*	*EF1α*[b]
Triticum aestivum	Bgta_A1	Belgium, Zwevegem	2009	JQ268160	JQ268188
	Bgta_A2	Belgium, Zwevegem	2009	JQ268114	JQ268209
	Bgta_A3	Belgium, Zwevegem	2009	JQ268096	JQ268196
	Bgta_A4	Belgium, Zwevegem	2009	JQ268095	JQ268213
	Bgta_A5	Belgium, Melle	2009	JQ268110	JQ268193
	Bgta_A6	Belgium, Melle	2009	JQ268116	JQ268216
	Bgta_A7	Belgium, Melle	2009	JQ268093	JQ268207
	Bgta_A8	Belgium, Melle	2009	JQ268121	JQ268204
	Bgta_A9	Belgium, Tongeren	2009	JQ268158	JQ268200
	Bgta_A10	Belgium, Tongeren	2010	JQ268111	JQ268212
	Bgta_A11	Belgium, Tongeren	2010	JQ268094	JQ268206
	Bgta_A12	Belgium, Tongeren	2010	JQ268098	JQ268202
	Bgta_A13	Belgium, Verrebroek	2009	JQ268118	JQ268225
	Bgta_A14	Belgium, Verrebroek	2009	JQ268092	JQ268210
	Bgta_A15	Belgium, Verrebroek	2009	JQ268106	JQ268221
	Bgta_A16	Belgium, Verrebroek	2009	JQ268107	JQ268224
	Bgta_A17	Belgium, Verrebroek	2010	JQ268112	JQ268194
	Bgta_A18	Belgium, Alveringem	2009	JQ268117	JQ268230
	Bgta_A19	Belgium, Bottelare	2010	JQ268099	JQ268195
	Bgta_A20	Belgium, Bottelare	2009	JQ268103	JQ268222
Triticale	BgTR_A21	Belgium, Melle	2009	JQ268126	JQ268171

	BgTR_A22	Belgium, Melle	2009	JQ268141	JQ268180
	BgTR_A23	Belgium, Melle	2009	JQ268144	JQ268164
	BgTR_A24	Belgium, Bocholt	2009	JQ268151	JQ268184
	BgTR_A25	Belgium, Bocholt	2010	JQ268137	JQ268191
	BgTR_A26	Belgium, Bocholt	2010	JQ268129	JQ268167
	BgTR_A27	Belgium, Bocholt	2010	JQ268153	JQ268190
	BgTR_A28	Belgium, Hoogstraten	2009	JQ268125	JQ268199
	BgTR_A29	Belgium, Hoogstraten	2009	JQ268163	-
	BgTR_A30	Belgium, Hoogstraten	2010	JQ268135	JQ268231
	BgTR_A31	Belgium, Hoogstraten	2010	JQ268152	JQ268166
	BgTR_A32	Belgium, Sint-Niklaas	2010	JQ268138	JQ268197
	BgTR_A33	Belgium, Sint-Niklaas	2010	JQ268123	JQ268170
	BgTR_A34	Belgium, Sint-Niklaas	2010	JQ268154	JQ268208
	BgTR_A35	Belgium, Bottelare	2010	JQ268133	JQ268182
	BgTR_A36	Belgium, Bottelare	2010	JQ268145	JQ268175
	BgTR_A37	Belgium, Kerkhove	2009	JQ268147	JQ268215
Secale cereale	BgS_A38	Belgium, Bottelare	2010	JQ268149	JQ268183
	BgS_A39	Belgium, Bottelare	2010	JQ268156	JQ268179
Triticum aestivum	Bgta_B1	France, Clermont-Ferrand	2009	JQ268120	JQ268198
	Bgta_B2	France, Bergerac	2009	JQ268127	JQ268165
	Bgta_B3	France, Cappelle-en-Pévèle	2010	JQ268108	JQ268223
	Bgta_B4	France, Cappelle-en-Pévèle	2010	JQ268136	JQ268174
	Bgta_B5	France, Cappelle-en-Pévèle	2010	JQ268161	-
	Bgta_B6	France, Cappelle-en-Pévèle	2010	JQ268102	JQ268172
	Bgta_B7	France, Cappelle-en-Pévèle	2010	JQ268128	JQ268181
Triticale	BgTR_B8	France, Clermont-Ferrand	2009	JQ268139	JQ268178

	BgTR_B9	France, Rennes	2009	JQ268157	-
	BgTR_B10	France, Cappelle-en-Pévèle	2010	JQ268132	JQ268169
	BgTR_B11	France, Cappelle-en-Pévèle	2010	JQ268134	JQ268168
	BgTR_B12	France, Cappelle-en-Pévèle	2010	JQ268122	JQ268173
	BgTR_B13	France, Drôme	2010	JQ268131	JQ268185
	BgTR_B14	France, Drôme	2010	JQ268155	JQ268177
	BgTR_B15	France, Druelle	2010	JQ268100	JQ268187
	BgTR_B16	France, Druelle	2010	JQ268097	JQ268186
Triticum aestivum	Bgta_C1	Poland, Bakow	2009	JQ268101	JQ268217
Triticale	BgTR_C2	Poland, Bakow	2009	JQ268124	JQ268220
	BgTR_C3	Poland, Uhnin	2009	JQ268162	JQ268227
	BgTR_C4	Poland, Bakow	2009	JQ268143	JQ268214
	BgTR_C5	Poland, Bakow	2009	JQ268142	JQ268176
	BgTR_C6	Poland, Bakow	2009	JQ268140	JQ268218
	BgTR_C7	Poland, Maslowice	2010	JQ268148	JQ268228
	BgTR_C8	Poland, Maslowice	2010	JQ268150	JQ268219
	BgTR_C9	Poland, Malyszyn	2010	JQ268146	JQ268211
	BgTR_C10	Poland, Karznicka	2010	JQ268130	JQ268229
Triticum aestivum	Bgta_D1	Israel, Be’eri	1980’s	JQ268115	JQ268203
	Bgta_D2	Israel, Yesodot	1980’s	JQ268109	JQ268201
	Bgta_D3	Israel, Nahal Oz	1980’s	JQ268119	JQ268205
Triticum durum	Bgtd_D4	Israel, Bet Dagan	1980’s	JQ268159	-
	Bgtd_D5	Israel, Hula	1980’s	JQ268113	JQ268189
	Bgtd_D6	Israel, Negba	1980’s	JQ268105	JQ268192
Triticum diccocoides	Bgtdic_D7	Israel, Ami’ad	1980’s	JQ268104	JQ268226

[a]All isolates below, until the next listed source host, were sampled on the same source host.

[b]We were unable to obtain *EF1α* sequences for the isolates BgTR_A29, Bgta_B5, BgTR_B9 and Bgtd_D4.
